# Dentists’ Dietary Perception and Practice Patterns in a Dental Practice-Based Research Network

**DOI:** 10.1371/journal.pone.0059615

**Published:** 2013-03-25

**Authors:** Yoko Yokoyama, Naoki Kakudate, Futoshi Sumida, Yuki Matsumoto, Gregg H. Gilbert, Valeria V. Gordan

**Affiliations:** 1 Research Fellow of the Japan Society for the Promotion of Science, Tokyo, Japan; 2 Department of Preventive Medicine and Epidemiology, National Cerebral and Cardiovascular Center, Suita, Osaka, Japan; 3 Stanford Prevention Research Center, Stanford University School of Medicine, Stanford, California, United States of America; 4 Graduate School of Medicine and Public Health, Kyoto University, Kyoto, Kyoto, Japan; 5 Nagayama Family Dental Clinic, Sapporo, Hokkaido, Japan; 6 Wing Town Dental Clinic, Okazaki, Aichi, Japan; 7 Department of Clinical and Community Sciences, School of Dentistry, University of Alabama at Birmingham, Birmingham, Alabama, United States of America; 8 Department of Restorative Dental Sciences at the University of Florida College of Dentistry, Gainesville, Florida, United States of America; University of Toronto, Canada

## Abstract

**Background:**

Dental caries are largely preventable, and epidemiological evidence for a relationship between diet and oral health is abundant. To date, however, dentists’ perceptions about the role of diet and dentists’ practice patterns regarding diet counseling have not been clarified.

**Objective:**

The purposes of this study were to: (1) examine discordance between dentists’ perception of the importance of diet in caries treatment planning and their actual provision of diet counseling to patients, and (2) identify dentists’ characteristics associated with their provision of diet counseling.

**Design:**

The study used a cross-sectional study design consisting of a questionnaire survey in Japan.

**Participants:**

The study queried dentists working in outpatient dental practices who were affiliated with the Dental Practice-Based Research Network Japan (JDPBRN), which aims to allow dentists to investigate research questions and share experiences and expertise (n = 282).

**Measurement:**

Dentists were asked about their perceptions on the importance of diet and their practice patterns regarding diet counseling, as well as patient, practice, and dentist background data.

**Results:**

The majority of participants (n = 116, 63%) recognized that diet is “more important” to oral health. However, among participants who think diet is “more important” (n = 116), only 48% (n = 56) provide diet counseling to more than 20% of their patients. Multiple logistic regression analysis suggested that several variables were associated with providing diet counseling; dentist gender, practice busyness, percentage of patients interested in caries prevention, caries risk assessment, and percentage of patients who receive blood pressure screening.

**Conclusions:**

Some discordance exists between dentists’ perception of the importance of diet in caries treatment planning and their actual practice pattern regarding diet counseling to patients. Reducing this discordance may require additional dentist education, including nutritional and systemic disease concepts; patient education to increase perception of the importance of caries prevention; or removing barriers to practices’ implementation of counseling.

**Trial Registration:**

ClinicalTrials.gov NCT01680848

## Introduction

Dental caries affect 60% to 90% of school children and almost 100% of adults, and is the most common chronic disease affecting children and adolescents [1], [2]. Oral health is essential to general health and quality of life, and the high prevalence of this disease highlights the importance of public health approaches to the prevention of dental caries [2].

Dental caries is largely preventable, and epidemiological evidence showing a relationship between diet and oral health is abundant [3], [4]. A previous study demonstrated that sugar intake reduction [5] and healthy eating patterns [6] are effective means of caries prevention. A healthy diet is also related to improvements in conditions such as hypertension [7], diabetes [8], cardiovascular disease [9], [10] and cancer [11], [12], [13]. The potential public health role of general dental practitioners in providing lifestyle advice to their patients has accordingly been recognized [14].

Previous studies have provided evidence that one-to-one dietary interventions in the dental setting can change the behavior of practitioners and patients [15]. The American Dietetic Association recommends collaboration between dietetics and dental professionals for oral health promotion and disease prevention and intervention [4]. Kakudate et al. showed that administering diet counseling was significantly associated with interproximal enamel surgical intervention thresholds; dentists who provide diet counseling tended not to intervene surgically into enamel carious lesions, an approach that is consistent with evidence-based dental treatment [16]. Kelly et al. pointed out that few dentists or other dental professionals conducted dietary counseling as a key component of regular patient care [17]. However, dentists’ dietary perceptions and practice patterns of diet counseling remain unclear.

The purposes of this study were to: (1) examine discordance between dentists’ perception of the importance of diet in caries treatment planning and their actual provision of diet counseling to patients, and (2) identify certain characteristics associated with their provision of diet counseling.

## Materials and Methods

### Study Design

We conducted a cross-sectional study consisting of a questionnaire survey in Japan between May 2011 and February 2012 [16]. This study was approved by the Ethics Committee of Kyoto University Graduate School and Faculty of Medicine (No. E1157). All participants provided written informed consent prior to participation in the study. We used the same questionnaire as that used in the US Dental Practice-Based Research Network (DPBRN) Study, “Assessment of Caries Diagnosis and Caries Treatment” [18], and the ‘DPBRN Enrollment Questionnaire’ [19]. Four dentists and clinical epidemiologists collaboratively translated these questionnaires into Japanese. The translated version of these questionnaires is available at http://www.dentalpbrn.org/uploadeddocs/Study%201(Japanese%20Version).pdf. (Original English version: http://nationaldentalpbrn.org/pdf/Study%201%20questionnaire%20FINAL%20after%20pre-testing%20021306.pdf). Dentists were asked about their perceptions on the importance of diet and their practice patterns regarding diet counseling, as well as patient, practice, and dentist background data.

The Dental Practice-Based Research Network Japan (JDPBRN) is a consortium of dental practices with a broad representation of practice types, treatment philosophies, and patient populations, and has a shared mission with the DPBRN [20], now called the National Dental PBRN (http://NationalDentalPBRN.org). The network regions of the JDPBRN represent all seven districts in Japan (Hokkaido, Tohoku, Kanto, Chubu, Kansai, Chugoku-Shikoku, and Kyushu). Questionnaires were distributed, answered, and returned by the method described in the previous study [16], [18], [21].

### Participants

We queried dentists working in outpatient dental practices who were affiliated with JDPBRN (n = 282). Participants who indicated that they perform restorative dentistry at their practice were recruited from the JDPBRN website and mailings.

### Dentists’ Dietary Perceptions and Practice Patterns of Diet Counseling

Perception of the importance of diet in a treatment plan was measured using the following question: For patients more than 18 years old, how important is diet when you decide on a treatment plan?: (1) not at all important, (2) slightly important, (3) moderately important, (4) very important, (5) extremely important.

Practice patterns of diet counseling were measured using the following question: What percentage of patients do you or your staff perform diet counseling for at some time while they are patients in your practice?

### Variable Selection

To identify dentist, patient, and practice characteristics associated with provision of diet counseling, theoretical models were discussed and identified in accordance with previous studies [16], [18], [22], [23]. In addition, explanatory variables were extracted, consisting of four categories: (1) dentists’ individual characteristics (years since graduation from dental school and gender), (2) practice setting (type of practice and busyness, and city population [government ordinance-designated city with population over 700,000 or not]), (3) patients’ characteristics (percentage of patients interested in caries prevention, patient age distribution, and percent of patients who self-pay), and (4) procedure-related characteristics (whether or not caries risk is assessed as a routine part of treatment planning, and percentage of patients receiving blood pressure screening).

### Statistical Analysis

#### 1. Description and relationship between dietary perception and practice patterns of diet counseling

We examined the relationship between perception of the importance of diet and practice patterns regarding diet counseling. Chi-square tests were performed to assess the association between perception and practice patterns.

Perception of the importance of diet was categorized dichotomously: “less important” [(1) not at all important, (2) slightly important, (3) moderately important], and “more important” [(4) very important, (5) extremely important] according to the previous study [24]. The percentage of patients reported by dentists who would receive diet counseling was reported as the mean score. For the assessment of robustness, we performed a sensitivity analysis using the median.

#### 2. Factors affecting the decision to provide diet counseling

Descriptive analysis was conducted via univariate regression analysis for explanatory variables associated with dentists’ practice patterns of diet counseling. Subsequently, multiple logistic regression analysis was conducted to examine the relationship between explanatory variables and prevalence of patients receiving diet counseling. Odds ratios and 95% confidence intervals (CIs) were calculated. All statistical analyses were performed with the STATA/SE® (version 10; STATA Corporation, College Station, TX, USA). Statistical significance was set at p<0.05.

## Results

### Demographic Information of Participants

Questionnaires were distributed to 282 dentists, and valid responses were collected from 189 (67%). Demographic characteristics of study participants are shown in Table 1 [16]. The mean number of years (±standard deviation) elapsed since graduation from dental school was 18.5±9.9, and participants were predominantly male (n = 154, 82%). With regard to practice setting, 40% (n = 76) of practices were established in government ordinance-designated cities of over 700,000. The percentage of dentists who perform caries risk assessment as a routine part of treatment planning was 26% (n = 49).

**Table 1:** Participant characteristics [16].

	Number (%) or Mean±SD
**Dentist's Individual Characteristics**	
Years since graduation from dental school (year)* (n = 185)	18.5±9.9
Gender (male), n(%) (n = 187)	154 (82)
**Practice Setting**	
Type of practice, n (%) (n = 182)	
Employed by another dentist	77 (41)
Self-employed without partners and without sharing of income, costs, or office space	105 (56)
Practice busyness, n (%) (n = 181)	
Too busy to treat all people requesting appointments	19 (11)
Provided care to all, but the practice was overburdened	72 (40)
Provided care to all, but the practice was not overburdened	59 (33)
Not busy enough	31 (17)
City population (government ordinance-designated city), n(%) (n = 189)	76 (40)
**Patients’ Characteristics**	
Percentage of patients interested in caries prevention, n(%) (n = 189)	
0% (none)	16 (8)
1–24%	80 (42)
25–49%	38 (20)
50–74%	46 (24)
75–99%	8 (4)
100%	1 (1)
Patient age distribution*	
1–18 years old (%) (n = 183)	16.1±13.2
19–44 years old (%) (n = 188)	24.8±11.0
45–64 years old (%) (n = 183)	30.4±11.2
65+ years (%) (n = 183)	28.5±17.4
Percent of patients who self-pay (%)*(n = 183)	8.6±16.6
**Dental Procedure Characteristics**	
Caries risk is assessed as a routine part of treatment planning, n(%) (n = 189)	49 (26)
Percentage of patients get blood pressure screening (%)*(n = 183)	9.7±20.9
Percentage of patients who receive diet counseling (%)*(n = 183)	21.4±27.2

* Mean±SD.

### Dentists’ Dietary Perceptions and Practice Patterns of Diet Counseling

**Figure 1:**
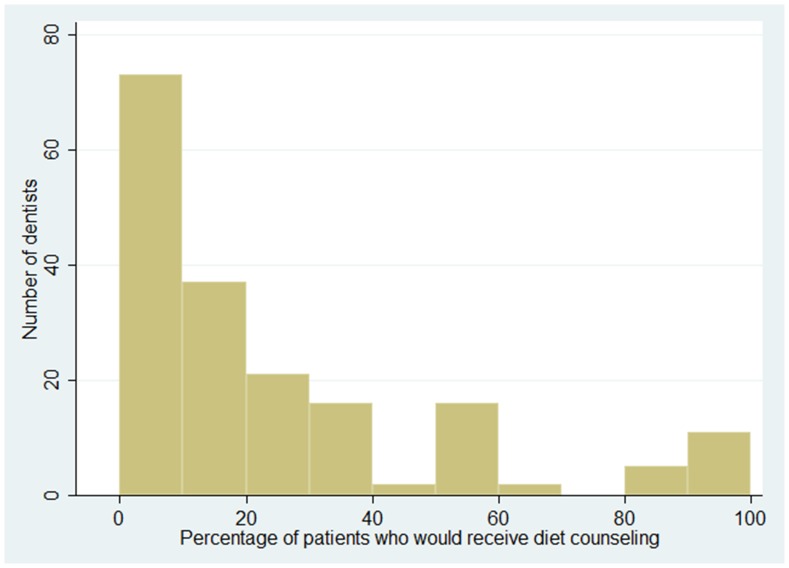
Distribution of the percentage of patients reported by dentists who would receive diet counseling. More than 50% of dentists reported that less than 20% of their patients would receive diet counseling.

One hundred and sixteen participants (63%) answered that they value diet as a “more important” aspect when they decide on a treatment plan (Table 2). The percentage of patients who would receive diet counseling is shown in Figure 1. The mean percentage of patients who would receive diet counseling was 21% (Table 1). Seventy-three participants (40%) gave diet counseling to more than 20% of patients (Table 2).

**Table 2:** Dentists' dietary perception and practice of diet counseling* (n = 183).

	Less important, n (%)	More important, n (%)	Total, n (%)
% of patients receiving diet counseling <20%	50 (75)	60 (52)	110 (60%)
% of patients receiving diet counseling ≥20%	17 (25)	56 (48)	73 (40%)

* p = 0.002.

Table 2 shows the relationship between participants’ dietary perceptions and practice patterns regarding diet counseling. Among participants who believe diet is “more important” (n = 116), only 48% (n = 56) gave diet counseling to more than 20% of their patients. Participants who think diet is “more important” gave diet counseling significantly more often than those who think diet is “less important” (p = 0.002) (Table 2).

### Factors Affecting the Decision to Perform Diet Counseling

The results of multiple logistic regression analysis are shown in Table 3. Five factors were significantly associated with the intent to provide diet counseling. The odds ratios (95% CI) were: gender, 2.76 (1.04–7.34); practice busyness, 0.21 (0.04–0.97); percentage of patients interested in caries prevention, 1.98 (1.31–3.01); doing caries risk assessment, 2.59 (1.02–6.59); and percentage of patients getting blood pressure screening, 1.24 (1.03–1.49). The results were similar when an analysis used an outcome cut-off of 15% (median) instead of 20% (mean).

**Table 3:** A multiple logistic regression of whether the dentist provides diet counseling on 20% or more of patients (n = 163).

Variable	Odds Ratio	95% CI		p value
**Dentists’ Individual Characteristics**				
Years since graduation from dental school	1.02	0.98	1.06	0.342
Gender (reference: male)	2.76	1.04	7.34	0.041
**Practice Setting**				
Type of practice				
Employed by another dentist	1.00			
Self-employed without partners and without sharing of income, costs, or office space	0.85	0.36	1.98	0.700
Practice busyness				
Too busy to treat all people requesting appointments	1.00			
Provided care to all who requested appointments, but the practice was overburdened	0.69	0.20	2.38	0.561
Provided care to all who requested appointments, but the practice was not overburdened	0.62	0.17	2.32	0.481
Not busy enough- the practice could have treated more patients	0.21	0.04	0.97	0.046
City population (reference: non-government ordinance designated city)	1.22	0.56	2.65	0.623
**Patients’ Characteristics**				
Percentage of patients interested in caries prevention	1.98	1.31	3.01	0.001
Percentage of elderly patients (65 or older)	1.00	0.98	1.02	0.902
Percentage of practice revenue or charges from self-pay	1.00	0.97	1.02	0.914
**Dental Procedure Characteristics**				
Caries risk assessment is done as a routine part of treatment planning (reference: no)	2.59	1.02	6.59	0.046
Percentage of patients receiving blood pressure screening (every 25%)	1.24	1.03	1.49	0.025

The outcome of interest (diet counseling) was coded 1 = provides diet counseling on 20% or more of the practice’s patients; 0 = does not. Hosmer-Lemshow goodness-of-fit, 0.91.

## Discussion

The majority of participants (n = 116, 63%) recognized that diet is “more important” when they decide on a caries treatment plan. However, less than half (n = 56, 48%) of these participants who indicated that diet is “more important” would give diet counseling to more than 20% of their patients. Results of multiple logistic regression analysis suggested that several variables were associated with dentists’ intent to give diet counseling. Specifically, dentist’s gender, practice busyness, percentage of patients interested in caries prevention, doing caries risk assessment, and percentage of patients receiving blood pressure screening, were all significantly associated with providing diet counseling.

The proportion of participants who place high value on the importance of diet for deciding a treatment plan (63%) was consistent with previous studies. According to the results of the same questionnaire survey by the US DPBRN, 67% of male and 72% of female dentists think patient’s diet is very or extremely important when they decide on a treatment plan [24]. Also, Kelly et al. noted that 66% of British dentists in their study responded that they believe that nutrition plays an important role in the maintenance of periodontal health [17]. These studies showed that over 60% of dentists in the US, UK, and Japan recognized the importance of diet.

However, only about 20% of patients would receive diet counseling in this study [16]. This low rate of dietary counseling practice patterns in dental clinics is consistent with previous studies. Kelly et al. also showed that only 14% of dentists or other dental professionals provided dietary advice as a regular part of patient care [17]. Touger-Decker et al. pointed out that perceived needs for nutrition education in dental school were high, with most respondents indicating the need for graduates of dental school programs to know how and when to conduct a nutrition assessment [25]. These findings indicate that achieving an increase in practice patterns of administering diet counseling in dental settings depends on the provision of appropriate education for dentists.

Our study clarified that female dentists and dentists who routinely do caries risk assessment tend to give more diet counseling than males or those who do not routinely do caries risk assessment. Also, according to the results of the same questionnaire survey by the US DPBRN, female dentists showed a greater overall preventive orientation than male dentists [24]. In addition, our previous results showed that female dentists and dentists who conduct caries risk assessment tend not to surgically intervene in enamel carious lesions, which is considered best practice for non-cavitated caries confined to enamel [16]. These results suggest that these factors are possibly related to overall preventive practices, including the provision of diet counseling.

Practice busyness was not a disincentive for participants’ decisions to give diet counseling. Our previous study clarified that practice busyness was not related to dental preventive practice, such as interproximal enamel surgical intervention thresholds [16]. This study shows that dentists who are not sufficiently busy did not tend to give more diet counseling than those who are busy. This result suggests that Japanese dentists do not decide whether or not to give diet counseling based on practice busyness.

Also, screening for high blood pressure (BP) was associated with administration of diet counseling. BP screening is particularly important for dentists to better manage patients with hypertension so that anxiety and pain can be avoided. More importantly BP should be carefully monitored and ideally be well under-control before the dentist begins certain dental treatments that have the potential to increase blood pressure [26]. In this study, 69 participants (38%) did not provide blood pressure screening to any patients. The safety and effectiveness of oral health care will be improved if more dentists provide blood pressure screening as a part of general preparation for the management of emergencies.

In addition, dentists’ perception of patient interest in preventive programs was associated with the provision of diet counseling in this study. Previous studies pointed out that patient willingness, liability, cost, and time were perceived barriers to the performance of medical screening in dental practice [27]. In the present study, over half of participants (51%, n = 96) thought that less than 25% of their patients were interested in preventive programs. However, a previous study of patient attitudes to preventive programs pointed out that the majority of patients were willing to have a dentist conduct screening for heart disease, high blood pressure, and diabetes [28]. Ongoing improvement in understanding of preventive care in dental practice requires education for both patients and dentists. Additionally, a better understanding of patients’ and dentists’ agreement on need for communication of preventive care could be further explored [29].

The main strength of this study was its relatively wide variety of participants, with respondents hailing from all over Japan. The age and gender distribution of this study sample was similar to the actual distribution of dentists in Japan [30], thereby enhancing the generalizability of the findings. However, the study results should be approached with caution. First, participants were not selected by random sampling, but rather by responding to the invitation to participate in the JDPBRN. Second, no objective standard for cut-off regarding an adequate prevalence of patients receiving diet counseling has been established, although we used prior planned cut-offs using mean and median for the assessment of robustness. Finally, given the cross-sectional nature of our study, causative relationships between factors and the provision of diet counseling were difficult to assess.

### Conclusion

Our study confirmed that discrepancies between the high level of perception of diet importance and practice patterns in the provision of diet counseling exist. Remedying this discrepancy requires additional dentist education, including nutritional and systemic disease concepts; patient education to increase perception of the importance of caries prevention; and programs that provide the opportunity to demonstrate the feasibility of delivering packaged diet counseling programs for implementation in dental clinical settings.
